# Supplementary material for: Klf5 acetylation regulates luminal differentiation of basal progenitors in prostate development and regeneration
Source: Nat Commun. 2020 Feb 21;11:997. doi: 10.1038/s41467-020-14737-8 (PMC7035357; doi:10.1038/s41467-020-14737-8)
Supplement: Supplementary file 1 — Supplementary information [file 41467_2020_14737_MOESM1_ESM.pdf]

SUPPLEMENTARY INFORMATION

Klf5 acetylation regulates luminal differentiation of basal progenitors in prostate development and regeneration

Zhang et al.

I. **SUPPLEMENTARY FIGURES**
II. **SUPPLEMENTARY TABLES**

I. SUPPLEMENTARY FIGURES

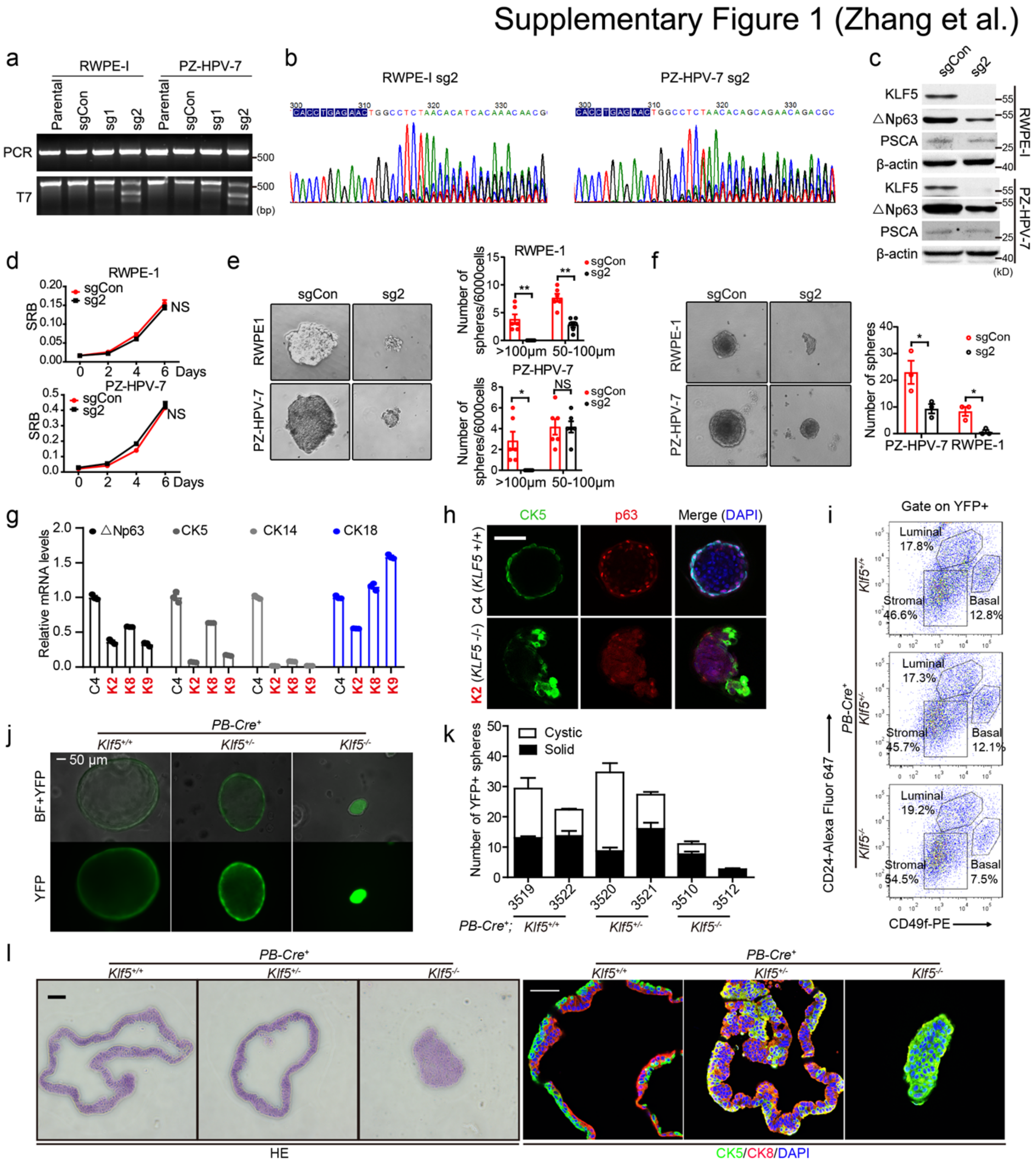

Supplementary Figure 1. KLF5 is indispensable for sphere and organoid forming capacities of human and mouse prostate epithelial cells respectively.

The CRISPR-Cas9 system was applied to both RWPE-1 and PZ-HPV-7 cell lines to eliminate endogenous *KLF5*. Sg1 and Sg2 are two sgRNAs designed to target the *KLF5* gene. Disruption of the *KLF5* gene by sgRNA-guided Cas9 was confirmed by both PCR (a) and DNA sequencing (b). Sg2 effectively reduced KLF5 expression, as measured by western blotting (c), which was accompanied by decreased expression of basal cell marker ΔNp63 (c). PSCA, a marker for transient amplifying (TA) cells in the prostate. (d, e) Deletion of *KLF5* did not affect cell proliferation, as measured by the SRB assay (d), but dramatically suppressed sphere formation in both Matrigel (e) and suspension culture (f). Data are shown in mean ± S.E.M. NS, not significant; *, $P<0.05$; **, $P<0.01$ (two-tailed Student's t-test). (g, h) Deletion of *KLF5* in RWPE-1 human prostate epithelial cells reduced the expression of basal cell markers CK5 and p63, as measured by realtime qPCR (g), and disturbed the organization of spheres, as indicated by IF staining (h). *KLF5*-null clones are marked in red. Real-time qPCR was performed in triplicate technically. S.E.M is plotted as error bar. (i-l) Deletion of *Klf5* in mouse prostatic epithelial cells, driven by PB-Cre and traced by YFP, reduced basal cells, as determined by flow cytometry (i); and impaired the organoid forming capability, as indicated by images (j), numbers (k) of representative organoids, and histological analysis of organoid structure by H&E staining and IF staining of basal marker CK5 and luminal marker CK8 (l). Representative images are selected based on the statistical analysis. Scale bar, 50 µm. Source data are provided as a Source Data file.

Supplementary Figure 2 (Zhang et al.)

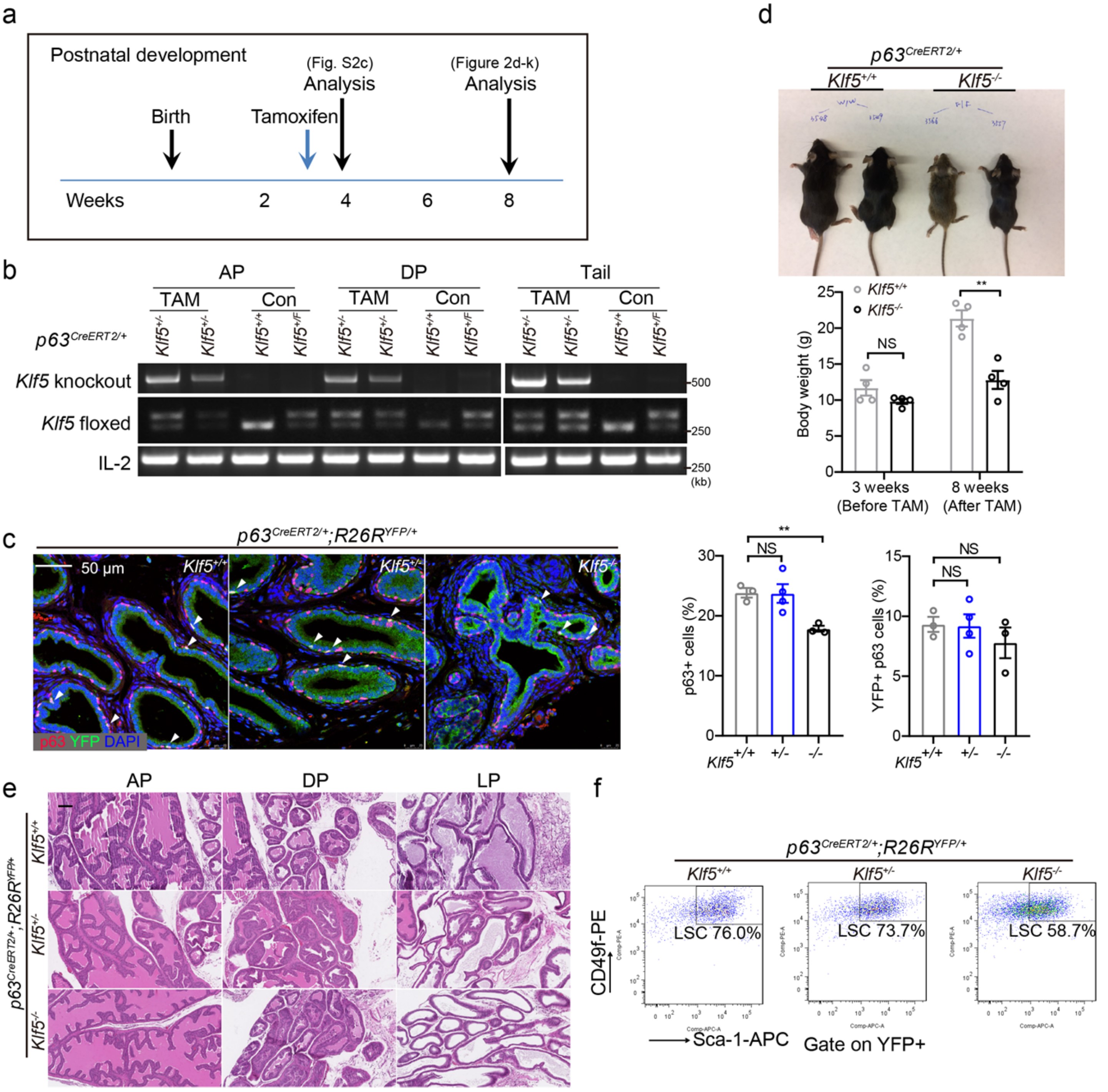

Supplementary Figure 2. Deletion of *Klf5* in basal cells decreases the body weight without altering morphology during postnatal development in mice.

(a) Schedule of tamoxifen administration for Cre induction and tissue collection. (b) Validation of *Klf5* deletion by PCR-based genotyping. IL-2 was used as an internal genotyping control. (c) Deletion of *Klf5* decreased p63+ cells but did not affect the YFP labeling efficiency after tamoxifen treatment, as detected by IF staining of YFP and basal marker p63 in the prostates of $p63^{CreERT2/+;}R26R^{YFP/+}$ mice with indicated *Klf5* genotypes. Tissues were collected immediately after the administration of tamoxifen. The numbers of mice are as follows: $Klf5^{+/+}$, n=3; $Klf5^{+/-}$, n=4; $Klf5^{-/-}$, n=3. Representative images are selected based on the statistical analysis. Scale bar, 50 µm.

(d) Basal cell-specific knockout of *Klf5* reduced the body weight of mice. Four mice were used for each group for statistical analyses. Data are shown in mean ± S.E.M. NS, not significant; **, P<0.01 (two-tailed Student's t-test). (e) Deletion of *Klf5* did not cause noticeable histological changes in mouse prostates, as analyzed by H&E staining. Scale bar, 50 µm. (f) Deletion of *Klf5* reduced the percentage of Lin-/Sca1+/CD49+ cells (LSC cells) in the prostate, as measured by flow cytometry. $Klf5^{+/+}$, wild-type *Klf5*; $Klf5^{+/-}$, hemizygous deletion of *Klf5*; $Klf5^{-/-}$, homozygous deletion of *Klf5*. Source data are provided as a Source Data file.

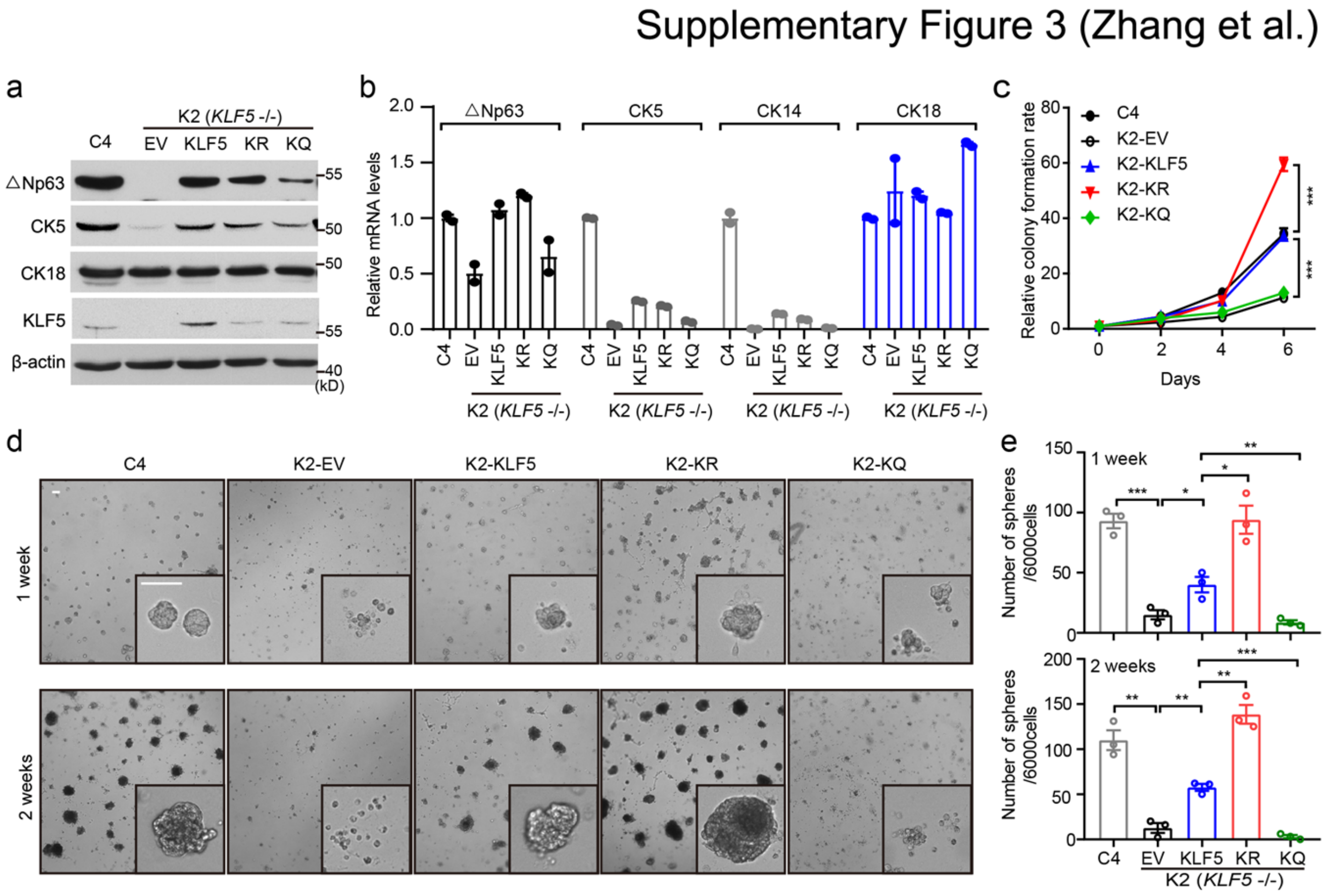

Supplementary Figure 3. Deacetylation of KLF5 promotes sphere formation of RWPE-1 cells.

(a, b) Deacetylation of KLF5 maintained but acetylation of KLF5 slightly compromised the expression of basal cell markers (ΔNp63, CK5 and CK14) while not significantly affecting the expression of luminal cell marker CK18, as detected by western blotting (a) and real-time qPCR (b). Wild-type *KLF5* (*KLF5*), acetylation-deficient mutant *KLF5* (*deAc-KLF5* or *KR*), a mutant mimicking acetylated *KLF5* (*Ac-KLF5* or *KQ*), and empty vector control (EV) were stably expressed in KLF5-null RWPE-1 cells, and C4 is a parental control clone. Real-time qPCR was performed in duplicate technically. (c) Deacetylation of KLF5 promoted, but acetylation of KLF5 attenuated, cell proliferation at a seeding density of 100 cells/ml in 2D culture, as indicated by the SRB assay. The day of seeding was defined as day one. (d, e) Sphere-forming capabilities of RWPE-1 cells were restored by KLF5, enhanced by KR (deAc-KLF5), but not improved by KQ (Ac-KLF5), as indicated by images (d) and numbers (e) of spheres. Representative images are selected based on the statistical analysis. Scale bars, 100 µm. Data are shown in mean ± S.E.M. *, P<0.05; **, P<0.01; ***, P<0.001 (two-tailed Student's t-test). Source data are provided as a Source Data file.

Supplementary Figure 4 (Zhang et al.)

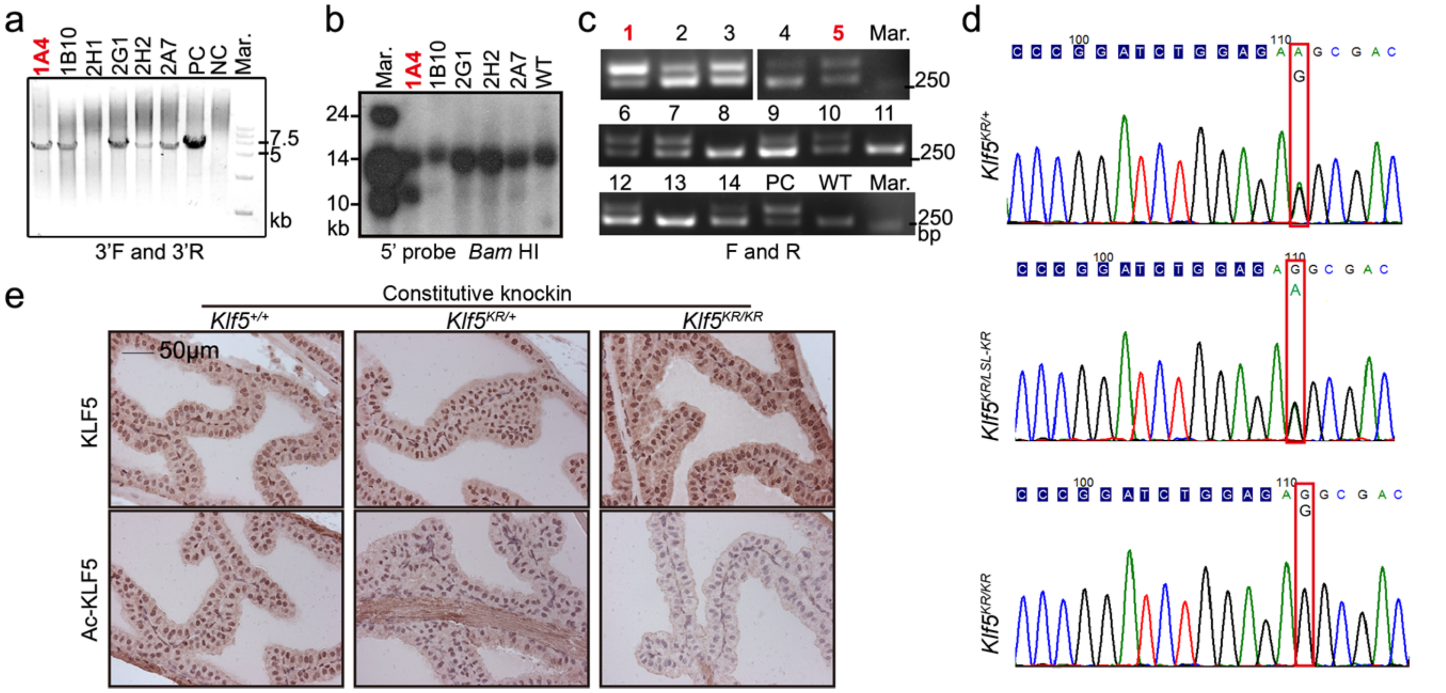

Supplementary Figure 4. Generation of *Klf5*^{K358R} mutant mice.

(a, b) Confirmation of successful targeting in embryonic stem cells (ESC) by long-amplifying PCR for the 3' arm using primers 3'F and 3'R (a) and by Southern blotting using the probe for the 5' arm (b). The 1A4 ESC clone, marked in red, was used for mouse production. Primers and probes were indicated in Figure 3a. (c) Detection of the *Klf5*^{KR} allele in chimeric mice by PCR using primers F and R in Figure 3a. Chimeric mice 1 and 5 were selected for further breeding. Mar, molecular weight marker. PC, positive control by using DNA of the 1A4 ESC clone. (d, e) The *Klf5*^{KR} knockin was confirmed at the both RNA level by cDNA sequencing (d) and protein level by IHC staining using anti-Ac-KLF5 antibody (e). Scale bar, 50 µm. Source data are provided as a Source Data file.

Supplementary Figure 5 (Zhang et al.)

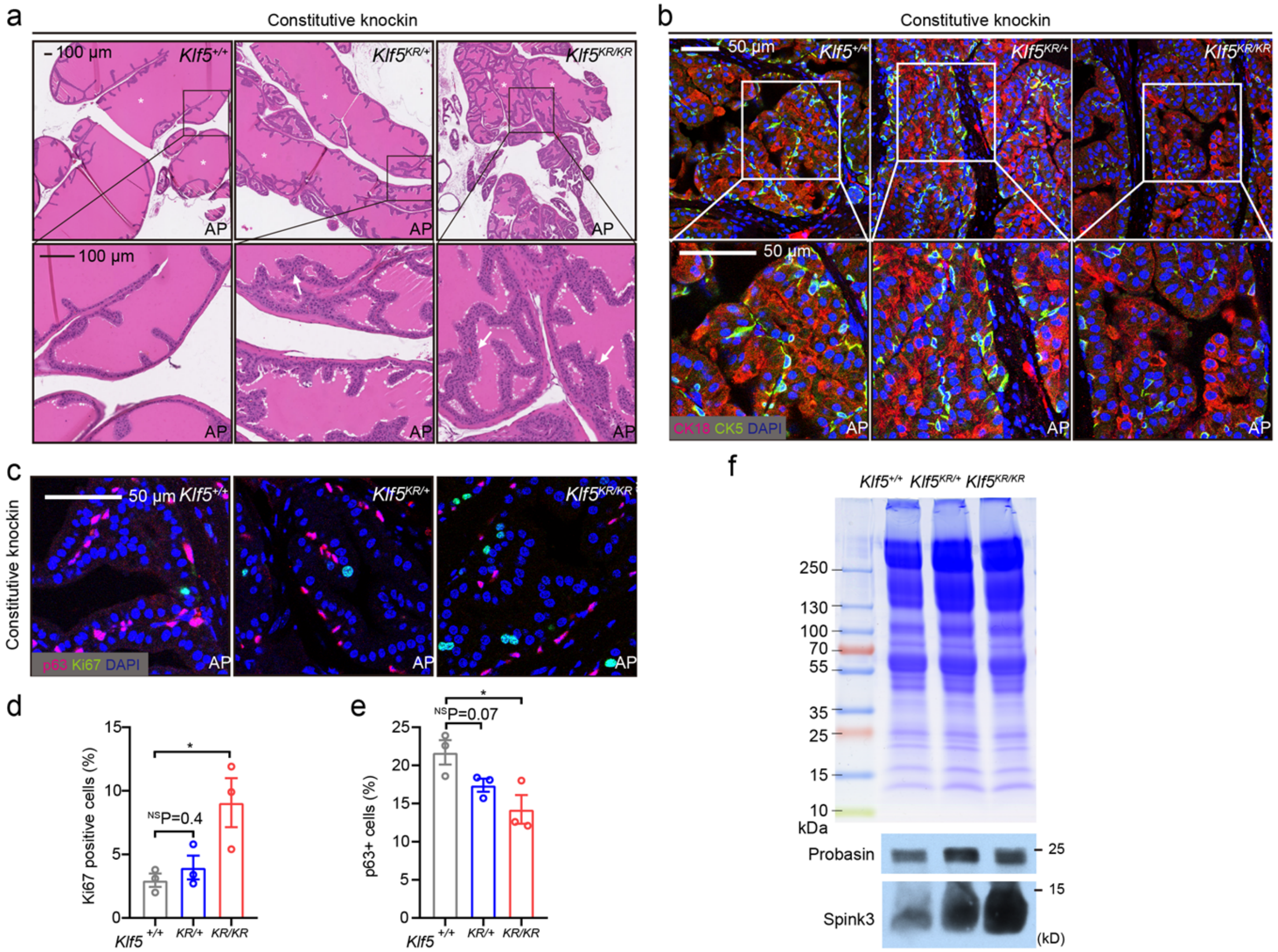

Supplementary Figure 5. Constitutive deacetylation of Klf5 reduces basal cells to attenuate postnatal prostate development in mice.

(a) Knockin of *Klf5*^{KR} retarded postnatal development of mouse prostates, as indicated by images of H&E stained 16-week old prostate sections where luminal areas are decreased (asterisks) and over-folded epithelia are visible (white arrows). (b) Knockin of *Klf5*^{KR} reduced basal cells in mouse prostates, as indicated by IF staining of markers for luminal (CK18) and basal (CK5) cells. (c-e) Knockin of *Klf5*^{KR} increased proliferating cells (c, d) but decreased basal cells (c, e) in the prostate, as indicated by the expression of Ki67 for cell proliferation and p63 for basal cells. (d) and (e) show 3 mice of each genotype. (f) Knockin of *Klf5*^{KR} did not affect the overall secretions in the lumen, while increasing two secretory proteins, probasin and Spink3, in secreted prostate fluid. The PBS-extracted proteins from the prostates of 8-week mice were separated on 5-20% gradient SDS-PAGE and stained with Coomassie Brilliant Blue G250 or subjected to western blotting. Representative images are selected based on the statistical analysis. Scale bars in a, 100 µm; scale bars in other panels, 50 µm. Data are shown in mean ± S.E.M. NS, not significant; *, P<0.05 (two-tailed Student's t-test). Source data are provided as a Source Data file.

Supplementary Figure 6 (Zhang et al.)

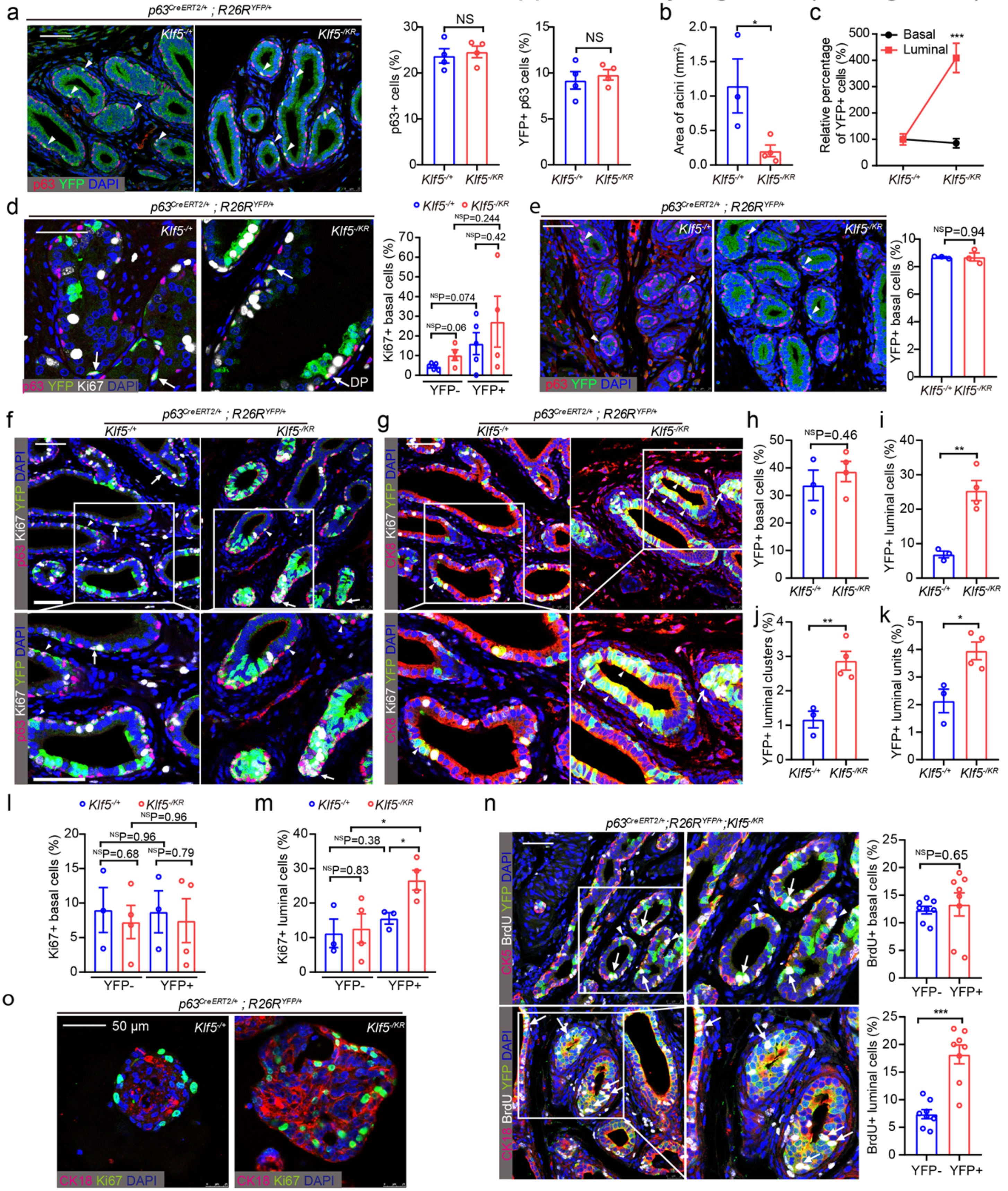

96

97 **Supplementary Figure 6. Induced *Klf5*^{K358R} knockin causes excess luminal differentiation of basal cells.**

(a) Knockin of $Klf5^{KR}$ by p63-Cre was induced by tamoxifen at postnatal day 18 for 5 consecutive days. Prostate tissues were collected immediately after tamoxifen administration for IF staining (a, left). The percentages of p63+ basal cells in total cells (a, middle) and YFP+ cells in basal cells (a, right) were calculated and statistically analyzed. Four mice were used for each genotype. (b) Knockin of $Klf5^{KR}$ decreased acinar areas of prostates. (c) Knockin of $Klf5^{KR}$ increased the relative percentage of YFP+ luminal cells but not that of basal cells. (d) Knockin of $Klf5^{KR}$ appeared to increase the proliferation index in basal cells but the increase was not statistically significant, as revealed by comparing cells stained for both the Ki67 proliferation marker and the p63 basal cell marker. (e-n) Knockin of $Klf5^{KR}$ by p63-Cre was induced by tamoxifen at postnatal day 7 for 5 consecutive days. (e) Prostate tissues were collected immediately after tamoxifen treatment for IF staining to determine the YFP-labeling efficiency. Three mice were used for each genotype. (f-m) Induced knockin of $Klf5^{KR}$ by tamoxifen at day 7 after birth in $p63^{CreERT2/+;}R26R^{YFP/+}$ mice did not affect the number of basal cells (f, h) but increased differentiated luminal cells (g, i) in the prostates of 6-week-old mice. IF staining was performed to detect YFP (green), basal marker p63 (red), luminal marker CK8 (red), and proliferation marker Ki67 (grey). The number of YFP+ luminal clusters (2 or more adjacent YFP+/CK8+ cells) was analyzed (j), and so was the number of YFP+ luminal units (single YFP+/CK8+ cells or cluster of YFP+/CK8+ cells) (k). Ki67 positive rate was calculated and statistically analyzed in both basal cells (l) and luminal cells (m). In h-m, the numbers of mice are as follows: $Klf5^{-/+}$, n=3; $Klf5^{-/KR}$, n=4. (n) The proliferation rate in the prostate of $p63^{CreERT2/+;}R26R^{YFP/+}; Klf5^{-/KR}$ mice was confirmed by costaining BrdU, YFP and basal cell marker CK5 or luminal cell marker CK18. BrdU was administrated at 100 mg/kg 16 hours before tissue collection. White arrows indicate YFP+/BrdU+ luminal cells. BrdU positive rates were statistically analyzed in the same pictures (n=8) from the same mouse prostate. (o) Knockin of $Klf5^{KR}$ increased proliferating luminal cells in organoids. Representative images are selected based on the statistical analysis. Scale bar, 50 µm. Data are shown in mean ± S.E.M. NS, not significant; *, P<0.05; **, P<0.01 (two-tailed Student's t-test). Source data are provided as a Source Data file.

Supplementary Figure 7 (Zhang et al.)

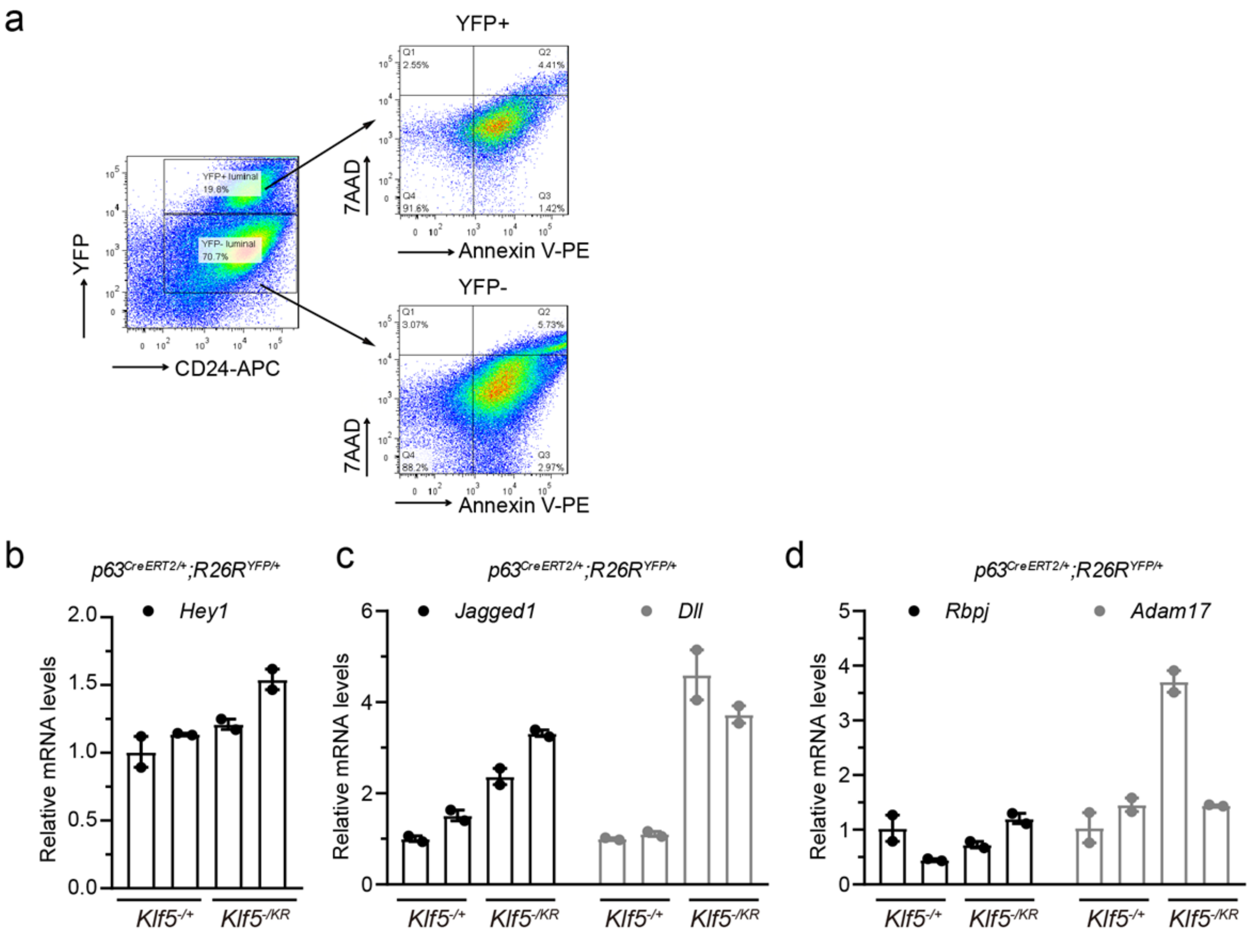

Supplementary Figure 7. Deacetylation of Klf5 upregulates multiple components of the Notch signaling pathway in mouse prostates.

(a) Gating strategies used for the analysis in Figure 5d. (b-d) Knockin of *Klf5*^{KR} in mouse prostates was induced by tamoxifen at day 18 after birth, and prostates were collected at postnatal week 8 for analysis. Real-time qPCR was used to detect the expression of Notch target gene *Hey1* (b), ligands *Jagged 1* and *Dll1* (c), processing machinery members Adam17 (d), and the Rbpj key transcription factor (d). *Klf5*$^{-/+}$ and *Klf5*$^{-/KR}$ indicate one wild-type *Klf5* allele and one *Klf5*^{KR} mutant allele respectively upon p63 promoter driven Cre expression. Real-time qPCR was performed in duplicate technically. S.E.M. is plotted as error bar. Source data are provided as a Source Data file.

Supplementary Figure 8 (Zhang et al.)

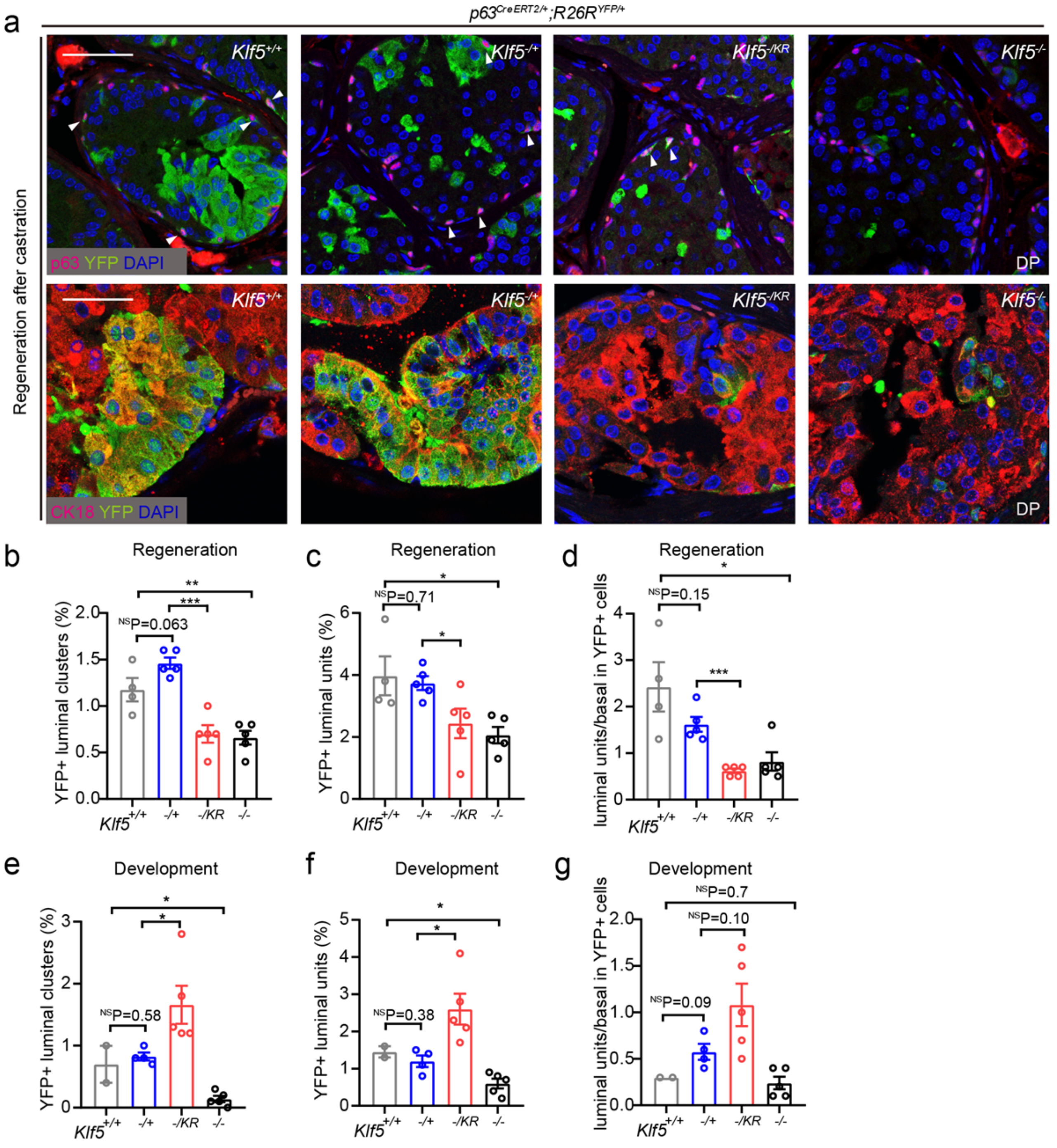

h

The percentage of YFP+ luminal cells in total luminal cells of mouse prostates

Group	Intact	Castrated	Regenerated
**WT-KLF5** (*Klf5*$^{-/+}$)	3.3% ± 0.70%	3.6% ± 0.48%	11.5% ± 1.17%
**DeAc-KLF5** (*Klf5*$^{-/KR}$)	13.6% ± 1.84%	0.4% ± 0.05%	4.5% ± 0.65%

Supplementary Figure 8. Induced deacetylation of Klf5 interferes with androgen-induced regeneration of basal progenitor-derived luminal cells in mouse prostates.

(a) Knockin of *Klf5*^{KR} in basal cells interfered with the regeneration of basal progenitor-derived luminal cells after a castration-regeneration cycle in dorsal prostates (DP), as indicated by IF staining for YFP, the p63 basal

cell marker, and the CK18 luminal cell marker in tissue sections. Representative images are selected based on the statistical analysis. Scale bars, 50 µm. Arrowheads and arrows indicate YFP-traced basal and luminal cells respectively. (b-d) Extended analyses of Figure 8a-d. Knockin of *Klf5*^{KR} dramatically reduced YFP+ luminal clusters (each cluster has 2 or more adjacent YFP+/CK18+ cells) (b), YFP+ luminal units (each unit is a single YFP+ luminal cell or a YFP+ luminal cluster) (c) and the ratio of YFP+ luminal units to basal cells (d) after one castration-regeneration cycle. (e-g) Extended analyses of Figure 8e-g. Knockin of *Klf5*^{KR} still increased YFP+ luminal clusters (e), YFP+ luminal units (f) and the ratio of YFP+ luminal units to basal cells (g) after 16-week normal development. (h) Summary of percentages of YFP+ luminal cells in total luminal cells of mouse prostates at indicated stages. WT-KLF5 and DeAc-KLF5 indicate *Klf5*$^{-/+}$ and *Klf5*$^{-/KR}$ of p63-Cre driven conditional knockout and knockin respectively. Data are shown in mean ± S.E.M. NS, not significant; *, P<0.05; **, P<0.01, ***, P<0.001 (two-tailed Student's t-test). Source data are provided as a Source Data file.

II. SUPPLEMENTARY TABLES

Supplementary Table 1. Genetic changes of *KLF5* alleles in RWPE-1 derived single clones.

Clone	Allele	Changes	Number	Stop Codon	Protein
**K2**	1	[Delete] TACAAATC	-8	Yes	No
	2	[Delete] TACAAATC	-8	Yes	
**K3**	1	[Delete] CAAATCCCA	-9	Yes	Yes
	2	[Change] TGGCCTCT->CAAATCCCA	+1	Yes	
**K6**	1	[Delete] CCTCTACAAATCCCAGAG	-18	No	Yes
	2	[Insert] CCTCTAACAAAAAT	+3	Yes	
**K8**	1	[Delete] CCTCTACAAATCCCAGAG	-4	Yes	No
	2	[Delete] CCTCTACAAATCCCAGAG	-1	Yes	
**K9**	1	[Delete] CCTCTACAAATCCCAGAG	-1	Yes	No
	2	[Delete] CCTCTACAAATCCCAGAG	-1	Yes	

Supplementary Table 2. Primer sequences used for genotyping.

Primer name	Sequences
Klf5-FKO For<br>(Klf5 FK358R For)	ACAGATTTGAGGCAGTTTGGC
Klf5-FKO Rev<br>(Klf5 FK358R Rev)	GGGCCAACTCCTAAGTGTTGC
Klf5-FK358R For	CGGATCGTTGAAGAAGGAGG
Cre For	CGGTCGATGCAACGAGTGAT
Cre Rev	CCACCGTCAGTACGTGAGAT
IL-2 For	CTAGGCCACAGAATTGAAAGATCT
IL-2 Rev	GTAGGTGGAAATTCTAGCATCATCC
Rosa-YFP Mutant	AAGACCGCGAAGAGTTTGTC
Rosa-YFP Common	AAAGTCGCTCTGAGTTGTTAT
Rosa-YFP Wild type	GGAGCGGGAGAAATGGATATG
P63-CreERT2 For	AATGTTGGGGTGTCTGGATG
P63-CreERT2 Rev-WT	CAGCAGTCAGGAACAAAGAGG
P63-CreERT2 Rev-KI	GCCCAAATGTTGCTGGATAG

Supplementary Table 3. Primers sequences used for realtime qPCR.

Primer name	Species	Primer sequences
dNP63-For	human	AGCCAGAAGAAAGGACAGCA
dNP63-Rev	human	TCACTAAATTGAGTCTGGGCAT
CK5-For	human	TGGTCTCCCGTGCCGCAGTTCTAT
CK5-Rev	human	ATTTGGGATTGGGGTGGGGATTCT
CK14-For	human	TGGCCGCGGATGACTTC
CK14-Rev	human	CTCGCTCTTGCCGCTCTG
CK18-For	human	CGCCAGGCCCAGGAGTATGAGG
CK18-Rev	human	ACTATCCGGCGGGTGGTGGTCTTT
GAPDH-For	human	GGTGGTCTCCTCTGACTTCAACA
GAPDH-Rev	human	GTTGCTGTAGCCAAATTCGTTGT
Tgf-β1-For	Mouse	CAACCCAGGTCCTTCCTAAA
Tgf-β1-Rev	Mouse	GGAGAGCCCTGGATACCAAC
Bmp4-For	Mouse	ATCAAACTAGCATGGCTCGC
Bmp4-Rev	Mouse	TGGACTGTTATTATGCCTTGTTTT
Bmp7-For	Mouse	CTTGGAAAGATCAAACCGGA
Bmp7-Rev	Mouse	GGACAGCCACTTCCTCACTG
Gli1-For	Mouse	ATTGGATTGAACATGGCGTC
Gli1-Rev	Mouse	GGATGAAGAAGCAGTTGGGA
Notch1-For	Mouse	CTGAGGCAAGGATTGGAGTC
Notch1-Rev	Mouse	GAATGGAGGTAGGTGCGAAG
Ptch1-For	Mouse	AATTCTCGACTCACTCGTCCA
Ptch1-Rev	Mouse	CTCCTCATATTTGGGGCCTT
Shh-For	Mouse	GGCCAAGGCATTTAACTTGT
Shh-Rev	Mouse	CCAATTACAACCCCGACATC
B-catenin-For	Mouse	CAGCTTGAGTAGCCATTGTCC
B-catenin-Rev	Mouse	GAGCCGTCAGTGCAGGAG
Fgf7-For	Mouse	CCCTTTGATTGCCACAATTC
Fgf7-Rev	Mouse	TTGACAAACGAGGCAAAGTG
Fgf10-For	Mouse	GTTGCTGTTGATGGCTTTGA
Fgf10-Rev	Mouse	GATTGAGAAGAACGGCAAGG
Fgfr2-For	Mouse	CGCTGTAAACCTTGCAGACA
Fgfr2-Rev	Mouse	CCTACCACCTGGATGTCGTT
FoxA1-For	Mouse	TGGTCATGGTGTTCATGGTC
FoxA1-rev	Mouse	GGAACAGCTACTACGCGGAC
Ar-For	Mouse	CGACTATTACTTTCCACCCCA
Ar-Rev	Mouse	TGCTGGCACATAGATACTTCTG
Nkx3.1 For	Mouse	CGACTGAACCCGAGTCTGAT
Nkx3.1 Rev	Mouse	ATGGCTGAACTTCCTCTCCA
Gapdh-For	Mouse	CCAGCCTCGTCCCGTAGACA
Gapdh-Rev	Mouse	GCCGTTGAATTTGCCGTGAG
Hes1-For	Mouse	GGCCTCTGAGCACAGAAAGT
Hes1-Rev	Mouse	GAATGCCGGGAGCTATCTTT
Myc-For	Mouse	AGTGCTGCATGAGGAGACAC
Myc-Rev	Mouse	GGTTTGCCTCTTCTCCACAG
