# Supplementary material for: Klf5 acetylation regulates luminal differentiation of basal progenitors in prostate development and regeneration
Source: Nat Commun. 2020 Feb 21;11:997. doi: 10.1038/s41467-020-14737-8 (PMC7035357; doi:10.1038/s41467-020-14737-8)
Supplement: Supplementary file 5 — Reporting Summary [file 41467_2020_14737_MOESM5_ESM.pdf]

nature research

Corresponding author(s): Jin-Tang Dong

Last updated by author(s): 12/02/2019

Reporting Summary

Nature Research wishes to improve the reproducibility of the work that we publish. This form provides structure for consistency and transparency in reporting. For further information on Nature Research policies, see Authors & Referees and the Editorial Policy Checklist.

Statistics

For all statistical analyses, confirm that the following items are present in the figure legend, table legend, main text, or Methods section.

n/a	Confirmed	
☐	☒	The exact sample size (*n*) for each experimental group/condition, given as a discrete number and unit of measurement
☐	☒	A statement on whether measurements were taken from distinct samples or whether the same sample was measured repeatedly
☐	☒	The statistical test(s) used AND whether they are one- or two-sided<br>*Only common tests should be described solely by name; describe more complex techniques in the Methods section.*
☒	☐	A description of all covariates tested
☐	☒	A description of any assumptions or corrections, such as tests of normality and adjustment for multiple comparisons
☐	☒	A full description of the statistical parameters including central tendency (e.g. means) or other basic estimates (e.g. regression coefficient) AND variation (e.g. standard deviation) or associated estimates of uncertainty (e.g. confidence intervals)
☐	☒	For null hypothesis testing, the test statistic (e.g. F, t, r) with confidence intervals, effect sizes, degrees of freedom and P value noted<br>*Give P values as exact values whenever suitable.*
☒	☐	For Bayesian analysis, information on the choice of priors and Markov chain Monte Carlo settings
☒	☐	For hierarchical and complex designs, identification of the appropriate level for tests and full reporting of outcomes
☒	☐	Estimates of effect sizes (e.g. Cohen's *d*, Pearson's *r*), indicating how they were calculated

Our web collection on statistics for biologists contains articles on many of the points above.

Software and code

Policy information about availability of computer code

Data collection	The numbers of cells labelled with indicated markers (eg, p63 labelled basal cells) were detected by using the Fiji software. Fiji is an upgrade version of Image J program, and available online at "https://imagej.net/Fiji". We used Plugins\Analyze\Cell counter for counting the cells with multiple colors, and used Analyze\Analyze particles to automatically count cells with single color.
Data analysis	GraphPad Prism 5 software, which is commercially available, was used for data analysis.

For manuscripts utilizing custom algorithms or software that are central to the research but not yet described in published literature, software must be made available to editors/reviewers. We strongly encourage code deposition in a community repository (e.g. GitHub). See the Nature Research guidelines for submitting code & software for further information.

Data

Policy information about availability of data

All manuscripts must include a data availability statement. This statement should provide the following information, where applicable:

- Accession codes, unique identifiers, or web links for publicly available datasets
- A list of figures that have associated raw data
- A description of any restrictions on data availability

The data generated and analyzed during the current study are available within the article as supplementary information, attached source data file, and from the corresponding author upon reasonable request.

Field-specific reporting

Please select the one below that is the best fit for your research. If you are not sure, read the appropriate sections before making your selection.

☒ Life sciences ☐ Behavioural & social sciences ☐ Ecological, evolutionary & environmental sciences

For a reference copy of the document with all sections, see nature.com/documents/nr-reporting-summary-flat.pdf

Life sciences study design

All studies must disclose on these points even when the disclosure is negative.

Sample size	Three to eight mice were included in each experimental group for determining the statistical significance.
Data exclusions	Some samples in the tissue microarray (TMA) were torn and/or generated a dark nonspecific background, and had to be excluded from analyses.
Replication	Experiments using cell lines were repeated at least twice, and consistent results were achieved.
Randomization	Pictures were taken from randomly selected fields.
Blinding	Quantification of IF data was done by a person who was blinded to genotypes and treatments of samples.

Reporting for specific materials, systems and methods

We require information from authors about some types of materials, experimental systems and methods used in many studies. Here, indicate whether each material, system or method listed is relevant to your study. If you are not sure if a list item applies to your research, read the appropriate section before selecting a response.

Materials & experimental systems

n/a	Involved in the study	
☐	☒	Antibodies
☐	☒	Eukaryotic cell lines
☒	☐	Palaeontology
☐	☒	Animals and other organisms
☒	☐	Human research participants
☒	☐	Clinical data

Methods

n/a	Involved in the study	
☒	☐	ChIP-seq
☐	☒	Flow cytometry
☒	☐	MRI-based neuroimaging

Antibodies

Antibodies used	All of the antibodies used in this study are described in the Methods section, including commercially available p63 (Biocare, CM163A); CK18 (Abcam, ab668); YFP (Abcam, ab13970); CK5 (Biolegend, PRB-160P); CK8 (Biolegend, MMS-162P); Ki67 (Thermo Fisher, RM-9106-S) and home-made antibodies against KLF5 and Ac-KLF5.
Validation	Validation information for all commercial antibodies are provided on the manufacturers' websites. The antibodies of KLF5 and Ac-KLF5 were generated and validated in our lab as described in our previous publications (Chen, C. et al. Oncogene 24:3319-27, 2005; Guo, P. et al. J Biol Chem, 284:6071-6078, 2009).

Eukaryotic cell lines

Policy information about cell lines

Cell line source(s)	RWPE-1 cell line was purchased from ATCC (Manassas, VA).
Authentication	ATCC provided a certificate.
Mycoplasma contamination	Confirmed to be free of contamination.
Commonly misidentified lines (See ICLAC register)	None

Animals and other organisms

Policy information about studies involving animals; ARRIVE guidelines recommended for reporting animal research

Laboratory animals	All of the mice used for analyses were male. The Klf5 knockout and KR knockin mice were in the C57BL/6 background. The p63-Cre mice were in 129 background. One- to 24- week mice were used for different analyses.
Wild animals	None
Field-collected samples	None
Ethics oversight	Use of mice at an Emory University Division of Animal Resources facility was approved by the Institutional Animal Care and Use Committee of Emory University.

Note that full information on the approval of the study protocol must also be provided in the manuscript.

Flow Cytometry

Plots

Confirm that:

☒ The axis labels state the marker and fluorochrome used (e.g. CD4-FITC).

☒ The axis scales are clearly visible. Include numbers along axes only for bottom left plot of group (a 'group' is an analysis of identical markers).

☒ All plots are contour plots with outliers or pseudocolor plots.

☒ A numerical value for number of cells or percentage (with statistics) is provided.

Methodology

Sample preparation	Described in the Methods section.
Instrument	Described in the Methods section.
Software	Described in the Methods section.
Cell population abundance	Cells were not sorted in the current study
Gating strategy	Described in the figure legends.

☒ Tick this box to confirm that a figure exemplifying the gating strategy is provided in the Supplementary Information.
