# Supplementary material for: Klf5 acetylation regulates luminal differentiation of basal progenitors in prostate development and regeneration
Source: Nat Commun. 2020 Feb 21;11:997. doi: 10.1038/s41467-020-14737-8 (PMC7035357; doi:10.1038/s41467-020-14737-8)
Supplement: Supplementary file 6 — Description of Additional Supplementary Files [file 41467_2020_14737_MOESM6_ESM.pdf]

Title: Supplementary Movie 1. Images of YFP-labeled organoids with *PB^{Cre};$Rosa^{YFP/+}$;$Klf5^{+/+}$*.
Description: Epithelial cells were isolated from 10-week old mouse prostates in which YFP labeling was driven by PB-Cre, and these cells were cultured for 7 days for organoid formation. Images were captured by confocal microscopy at different layers; and the images were then converted into movies.

Title: Supplementary Movie 2. Images of YFP-labeled organoids with *PB^{Cre};$Rosa^{YFP/+}$;$Klf5^{+/-}$*.
Description: Epithelial cells were isolated from 10-week old mouse prostates in which hemizygous deletion of Klf5 was driven by PB-Cre and traced by YFP expression, and these cells were cultured for 7 days for organoid formation. Images of YFP-labeled organoids were captured by confocal microscopy at different layers; and the images were then converted into movies.

Title: Supplementary Movie 3. Images of YFP-labeled organoids with *PB^{Cre};$Rosa^{YFP/+}$;$Klf5^{-/-}$*.
Description: Epithelial cells were isolated from 10-week old mouse prostates in which homozygous deletion of Klf5 was driven by PB-Cre and traced by YFP expression, and these cells were cultured for 7 days for organoid formation. Images of YFP-labeled organoids were captured by confocal microscopy at different layers; and the images were then converted into movies.
